# An Origami Paper-Based Biosensor for Allergen Detection by Chemiluminescence Immunoassay on Magnetic Microbeads

**DOI:** 10.3390/bios12100825

**Published:** 2022-10-04

**Authors:** Elisa Lazzarini, Andrea Pace, Ilaria Trozzi, Martina Zangheri, Massimo Guardigli, Donato Calabria, Mara Mirasoli

**Affiliations:** 1 Department of Chemistry “Giacomo Ciamician”, Alma Mater Studiorum, University of Bologna, Via Francesco Selmi 2, I-40126 Bologna, Italy; 2 Interdepartmental Centre for Industrial Agrofood Research (CIRI AGRO), Alma Mater Studiorum, University of Bologna, Via Quinto Bucci 336, I-47521 Cesena, Italy; 3 Interdepartmental Centre for Industrial Research in Advanced Mechanical Engineering Applications and Materials Technology (CIRI MAM), Alma Mater Studiorum, University of Bologna, Viale Risorgimento 2, I-40136 Bologna, Italy; 4 Interdepartmental Centre for Industrial Research in Renewable Resources, Environment, Sea and Energy (CIRI FRAME), Alma Mater Studiorum, University of Bologna, Via Sant’Alberto 163, I-48123 Ravenna, Italy; 5 Interdepartmental Centre for Industrial Aerospace Research (CIRI AEROSPACE), Alma Mater Studiorum, University of Bologna, Via Baldassarre Canaccini 12, I-47121 Forlì, Italy

**Keywords:** chemiluminescence, immunoassay, magnetic beads, ovalbumin, paper-based biosensors, origami

## Abstract

Food allergies are adverse health effects that arise from specific immune responses, occurring upon exposure to given foods, even if present in traces. Egg allergy is one of the most common food allergies, mainly caused by egg white proteins, with ovalbumin being the most abundant. As allergens can also be present in foodstuff due to unintended contamination, there is a need for analytical tools that are able to rapidly detect allergens in food products at the point-of-use. Herein, we report an origami paper-based device for detecting ovalbumin in food samples, based on a competitive immunoassay with chemiluminescence detection. In this biosensor, magnetic microbeads have been employed for easy and efficient immobilization of ovalbumin on paper. Immobilized ovalbumin competes with the ovalbumin present in the sample for a limited amount of enzyme-labelled anti-ovalbumin antibody. By exploiting the origami approach, a multistep analytical procedure could be performed using reagents preloaded on paper layers, thus providing a ready-to-use immunosensing platform. The assay provided a limit of detection (LOD) of about 1 ng mL^−1^ for ovalbumin and, when tested on ovalbumin-spiked food matrices (chocolate chip cookies), demonstrated good assay specificity and accuracy, as compared with a commercial immunoassay kit.

## 1. Introduction

Adverse reactions to food can be classified as food allergies and intolerances. According to the Expert Panel Report sponsored by the National Institute of Allergy and Infectious Diseases (NIAID), food allergy is defined as “an adverse health effect arising from a specific immune response that occurs reproducibly on exposure to a given food”, while food intolerance consists of “nonimmune reactions that include metabolic, toxic, pharmacologic, and undefined mechanisms”. In contrast to food intolerance, allergy is dose independent; thus, even the presence of traces of allergens can cause serious consequences [1]. As an effective treatment for food allergies is not yet available, allergen-suspicious food avoidance by sensitive consumers is the only possible strategy to avoid negative outcomes. Presently, no regulatory threshold exists for allergenic contents in food samples; therefore, highly sensitive analytical methods are required [2]. Egg allergy is one of the most common allergies, especially among infants and children, and can cause symptoms involving the respiratory system, or even potentially fatal anaphylaxis [3]. The egg white proteins ovalbumin, lysozyme, ovomucoid and ovotransferrin are the major egg allergens. Ovalbumin (OVA) is the most abundant, making up approximately 54% of the total albumen proteins [4], and is, therefore, considered as the best detection target for the search of egg allergens [5]. OVA is a phospho-glycoprotein of about 45 kDa, composed of 385 amino acids. OVA is not only present in food products that contain eggs, but it can be found in other food commodities. For example, it is used in the wine clarification process to eliminate the excess suspended matter, without altering the character of the drink. Ovalbumin is also used in many food products as an emulsifying agent or to bind ingredients together, thanks to its ability to thermally coagulate. Furthermore, the widespread use of OVA poses significant risks of unintended food contamination during food processing procedures. All these considerations call for the development of analytical tools that are able to rapidly detect OVA in a variety of products for human use.

The detection of allergens in food products can be pursued through different analytical approaches, the main being immunoanalytical, mass-spectrometry, nucleic-acid-based methods, and biosensors [6,7]. Laboratory methods are generally very sensitive and accurate; however, they are often laborious, time-consuming, expensive, and require experienced personnel and well-equipped laboratories. To enable fast and convenient point-of-use analyses, in recent decades, many efforts have been directed towards the development of simple-to-use devices for on-site analysis, thus allowing real-time measurement of allergens [2,8,9].

The conventional enzyme-linked immunosorbent assays (ELISAs), which employ antibodies for the highly specific analyte recognition and enzyme catalysts for sensitive detection, provide good analytical performance, especially when coupled with chemiluminescence (CL) detection [10]. However, they are not suited for point-of-use applications, as they need a large volume of reagents, time-consuming manipulations, and tedious washing steps. Lateral flow immunochromatographic assays (LFIAs) are routinely used for the on-site detection of allergen traces in finished products, due to their ease of use and competitive prices. However, most of them rely on colorimetric detection, exploiting colloidal gold as a tracer; thus, they only provide qualitative yes/no results [11,12].

Microfluidic paper-based analytical devices (µPADs) have recently attracted much attention due to their ease of use, low consumption of reagents, low cost, rapidity, portability, and disposability [13,14,15,16]. These devices use paper as a substrate to create microfluidic structures (e.g., channels, reagent mixers, reaction chambers) by patterning hydrophobic materials on hydrophilic paper. The use of origami (paper folding) and kirigami (paper cutting) techniques in the fabrication of µPADs has given researchers the opportunity to fabricate 3D paper-based devices, which provide high flexibility of application and allow us to conduct complex multistep analytical procedures, such as full immunoassays, on paper [17,18,19,20]. Indeed, in contrast to the simplest µPADs, such as LFIAs, in which fluids are only drawn horizontally through the device, 3D devices sustain the flow both horizontally and vertically. In addition to higher versatility, these devices often possess superior analytical performance, since the vertical flow provides higher assay rapidity and sensitivity [21]. Coupling this format with CL detection also takes advantage of CL’s specific features [22], such as amenability to miniaturization as well as high sensitivity and specificity of detection, even though only a few examples have been published up to now [23,24,25,26]. Furthermore, µPAD-based assays enable the immobilization of biospecific recognition molecules (such as enzymes, antigens, antibodies, aptamers, or nucleic acids) on paper. Bioprobes can be directly immobilized onto paper surfaces; however, this has the limitations of providing a limited surface area for the subsequent reactions and poor coating efficiency, and requiring complex procedures for substrate modification and surface functionalization [21]. On the contrary, particle-modified μPADs allow easy and efficient biomolecule immobilization in defined device areas, therefore improving the overall assay performance [27,28,29]. However, previously published particle-modified μPADs involve complex analytical protocols and in most cases, only the final signal detection is performed in the μPAD, thus compromising their amenability for point-of-use application.

Herein, we report an origami paper-based device, which employs magnetic microbeads (MBs) for paper substrate functionalization and is used to perform a competitive CL immunoassay for OVA in food samples. In this assay, OVA in the sample competes with OVA immobilized on MBs for a limited amount of HRP-labelled anti-OVA antibody (anti-OVA-HRP). The use of MBs enables easy and efficient OVA immobilization, as well as the production of a device in which the MBs are contained in well-defined reaction areas on paper, providing an increased surface area for immunoreaction. By exploiting the origami approach, all the steps of the immunoassay procedure (i.e., immunoreaction, washing, detection) were carried out by appropriately folding/unfolding the device. All the reagents required for assay execution are preloaded in dried form in the μPAD, so that only sample and buffer applications were required to complete the assay, with no need for handling chemicals or conducting complex procedures. Finally, as the washing step is critical for obtaining accurate analyte detection in immunoassays and because effective washing is quite challenging in µPAD-based formats, we designed and implemented multiple washing layers in the µPAD to solve this issue.

## 2. Materials and Methods

### 2.1. Chemicals

SPHERO^TM^ Carboxyl magnetic particles (MBs, 2.5% *w*/*v*, 3.90 µm diameter) were obtained from Spherotech Inc (Lake Forest, IL, USA). Ovalbumin from chicken egg white (lyophilized powder, ≥98%), bovine serum albumin (lyophilized powder, ≥96%), rabbit serum albumin (lyophilized power, ≥99%), lysozyme from chicken egg white (lyophilized powder, protein ≥90%), horseradish peroxidase (HRP, lyophilized powder, ≥250 U mg^−1^), casein from bovine milk (purified powder), N-hydroxysulfosuccinimide sodium salt (sulfo-NHS, ≥98%), 1-ethyl-3-(3-dimethylaminopropyl)carbodiimide hydrochloride (EDC, ≥98%), sorbitol (≥99%), 2-(N-morpholino)ethanesulfonic acid (MES, ≥99%), tris(hydroxymethyl)aminomethane (TRIS, ≥99.8%) and Tween 20 were purchased from Sigma-Aldrich (St Louis, MO, USA). HRP-labelled anti-ovalbumin polyclonal rabbit antibody (anti-OVA-HRP) and SuperSignal^TM^ ELISA Femto Maximum Sensitivity Substrate (a two-component luminol-based CL cocktail, composed of a luminol/enhancer solution and a stable peroxide solution) were obtained from Thermo-Fisher Scientific (Fair Lawn, NJ, USA). All the other chemicals were of the highest purity available. The Whatman CHR 1 chromatographic paper (200 × 200 mm sheets) was bought from Sigma-Aldrich.

The following buffers were used in the functionalization of MBs and in the assay procedure: PBS (10 mmol L^−1^ phosphate buffer, pH 7.4, containing 137 mmol L^−1^ NaCl), PBST (PBS containing 0.05% *v*/*v* Tween 20), MES (25 mmol L^−1^ MES, pH 5.0), and TRIS (25 mmol L^−1^ TRIS, pH 7.0, containing 250 mmol L^−1^ NaCl).

For assay validation, a commercial colorimetric microtiter plate-based ELISA kit for the quantitative detection of OVA in food samples (AgraQuant^®^ Ovalbumin, Romer Labs Division Holding GmbH, Getzersdorf, Austria) has been used. Samples have been extracted following the procedure described in Section 2.6 and assayed according to the manufacturer’s instructions.

### 2.2. Functionalization of Magnetic Microbeads

A volume of 170 µL of carboxyl MBs stock suspension (2.5% *w*/*v*) was transferred into a tube and washed twice for 10 min with 1400 µL of PBS. Surface carboxyl groups were activated by incubating the MBs for 35 min with 700 µL of an EDC/sulfo-NHS solution (100 µg mL^−1^ each in MES buffer). The supernatant was then removed and the MBs were washed twice for 10 min with 1400 µL of PBS. The activated MBs were incubated overnight with 700 µL of an OVA solution (10 µg mL^−1^ in PBS). After washing for 10 min with 1400 µL of PBST, unreacted surface groups were deactivated by incubating the MBs for 60 min with 700 µL of a 1.0 mol L^−1^ ethanolamine solution in PBS. The OVA-MBs were then washed for 10 min with 1400 µL of PBS and incubated for 10 min with 1400 µL of a casein solution (1% *w*/*v* in PBS), to avoid non-specific adsorption of proteins on the surface of MBs. Upon washing with 1400 µL of PBS, OVA-MBs were resuspended in 1200 µL of PBS to achieve a final concentration of about 3.5 mg mL^−1^ and stored at 4 °C until use.

All incubation and washing steps were performed at room temperature, under mild shaking in 12 × 75 mm borosilicate glass tubes. A home-made tube rack equipped with NdFeB magnets was used in the washing steps to capture the MBs before removal of the supernatant.

### 2.3. Fabrication of the Origami µPAD Device

**Figure 1:**
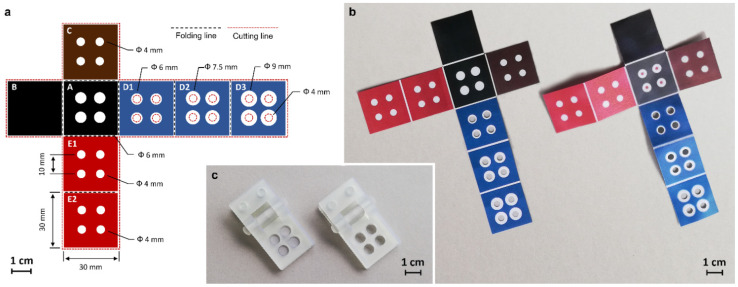
(**a**) Design of the hydrophobic areas of the origami µPAD. Black and red dashed lines represent folding lines (created by a manual rotary perforating blade) and cutting lines, respectively. A: base layer; B: anti-leaching layer; C: immunoreaction layer; D: washing layers; E: CL detection layers. (**b**) Images of the origami µPAD just after cutting of excess paper (**left**) and upon loading and air-drying of reagents and OVA-MBs (**right**). (**c**) Images of the spring-loaded 3D-printed holding clips one equipped with magnets and used for loading of OVA-MBs in the µPAD (**left**) and one without magnets and employed in the assay procedure (**right**). The scale bars represent 1 cm.

The origami µPAD was produced by drawing the layout of the hydrophobic areas on PowerPoint (Figure 1a) and printing the areas on a 200 × 200 mm Whatman CHR 1 chromatography paper sheet using a commercial solid ink Phaser 8560DN printer (Xerox Co., Norwalk, CN, USA). The folding lines were created by a manual rotary perforating blade and the µPAD was cut from the paper sheet and heated at 120 °C for 10 min in an oven to melt the wax-based solid ink, which diffused into the paper, generating the hydrophobic barriers. Then, the reagents were loaded into the origami µPAD by dispensing the solutions onto the four hydrophilic areas of levels A (first 10 µL of 3.5 mg mL^−1^ OVA-MBs suspension in PBS was added in each area and then, after drying, 15 µL of 1% *w*/*v* casein solution in PBS was added to saturate the paper surface), C (5 µL of 1 µg mL^−1^ anti-OVA-HRP conjugate solution in PBS containing 1 mg mL^−1^ sorbitol in each area), E1 (20 µL of the luminol/enhancer solution of the SuperSignal^TM^ substrate in each area), and E2 (20 µL of 10 mmol L^−1^ sodium perborate solution in PBS in each area); the solutions in layers E1 and E2 were loaded through four successive 5-µL additions, each after complete evaporation of the liquid. After air-drying at room temperature in the dark for 1 h, the biosensor was vacuum sealed in a plastic bag and stored at 4 °C and in the dark until use (Figure 1b).

To facilitate the preparation of the biosensor and the assay procedure, we used two spring-loaded holding clips (Figure 1c). The clips were designed and produced in clear resin by stereolithography (SLA) 3D-printing with a Form 2 desktop 3D printer (Formlabs Inc, Somerville, MA, USA). One clip, equipped with four small NdFeB magnets (N45 grade, 8 mm diameter, 3 mm height) located in the bottom half in the correspondence of the four hydrophilic areas of the µPAD, was used during the loading of OVA-MBs to avoid their excessive dispersion over the hydrophilic area. The second one had four holes in both halves and was used in the assay procedure to guarantee the contact between the layers in the folded origami µPAD, still permitting the addition of buffers and imaging of the CL signal.

### 2.4. Assay Procedure

**Figure 2:**
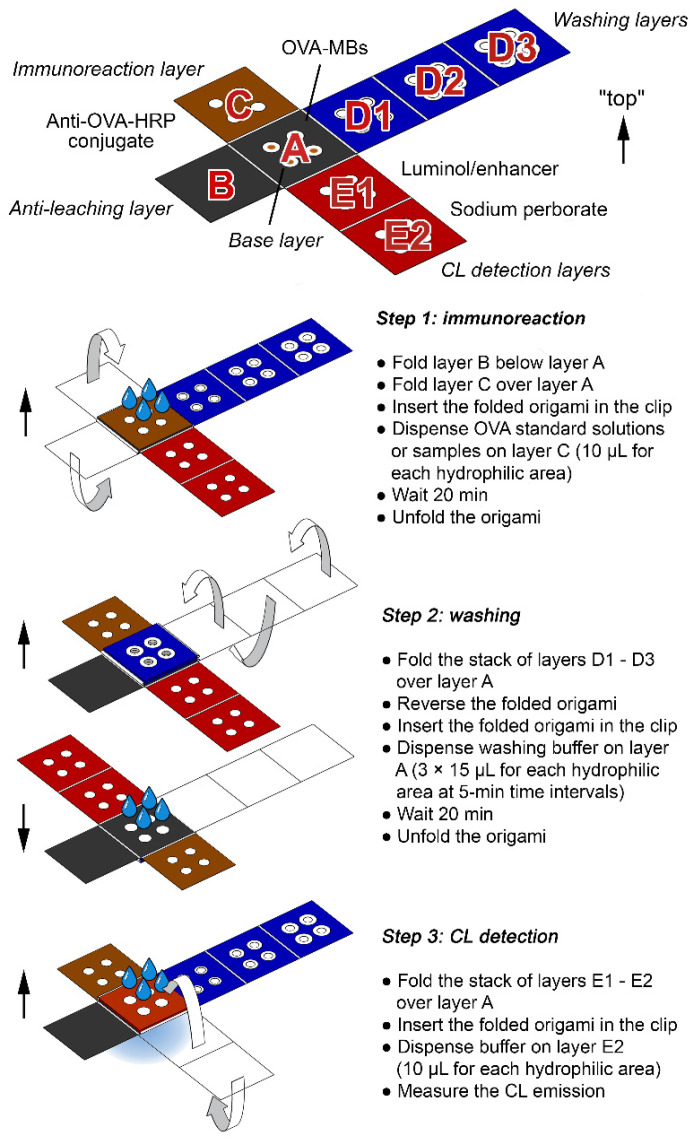
Scheme of the analytical procedure for the determination of OVA using the origami µPAD. In each assay step, upon folding, the origami µPAD was inserted into the 3D-printed holding clip (not shown).

The overall assay procedure is outlined in Figure 2 and shown in the Supplementary Materials (Video S1: Assay procedure). The origami µPAD was removed from the sealed plastic bag. To configure the origami for the first assay step, layer C was folded over layer A and layer B was folded under layer A. The folded origami was inserted in the holding clip with layer C upwards, then 10 µL of the solutions to be assayed, namely OVA-free solution (PBS), low (0.003 µg mL^−1^) and high (1 µg mL^−1^) OVA standard solutions in PBS, and the sample, was deposited on each hydrophilic area of layer C to solubilize the anti-OVA-HRP conjugate and start the immunological reaction. Upon 20 min of incubation at room temperature, the origami was unfolded, then the stack of layers D1–D3 was folded over layer A. The folded origami was inserted in the holding clip with layer A upwards and three 15 µL-aliquots of washing buffer were deposited at 5 min-time intervals on each hydrophilic area of layer A to remove all unbound species from the MBs in this layer. Finally, after 20 min, the origami was unfolded and the stack of layers E1–E2 was folded over layer A. The folded origami was inserted in the holding clip with layer E2 upwards and 10 µL of PBS was added on each hydrophilic area of layer E2, to dissolve the components of the luminol-based CL cocktail required to perform CL detection of the anti-OVA-HRP conjugate bound to the MBs. The CL emission produced by the MBs in layer A was then measured, employing a portable, battery-operated, two-stage Peltier cooled charge coupled device (CCD) camera (ATIK 11000, ATIK Cameras, New Road, Norwich) adapted to perform contact imaging detection, as previously described [30]. A sequence of 100 consecutive images with exposure time of 15 s was acquired, starting immediately after the addition of the buffer. The CL images were analyzed using the freeware ImageJ v.1.53h software (National Institutes of Health, Bethesda, MD). Regions of interest (ROIs) corresponding to the four OVA-MBs deposition areas of layer A were defined and for each image, the CL emission intensities were evaluated by integrating the CL emissions on the ROI areas. Finally, the analytical CL signals were obtained by reconstructing the CL emission intensity kinetic profiles and evaluating the total CL emission as the area under the curve. The ratios between the CL signals of the OVA standards or of the sample and the CL signal of the OVA-free solution were calculated. Finally, the logit of the CL signal ratios of the two OVA standards was plotted against the logarithm of OVA concentration to obtain a two-point linear calibration curve and the amount of OVA in the unknown sample was evaluated by interpolation of its CL signal ratio logit on the calibration curve.

### 2.5. Data Elaboration and Statistics

All measurements were performed at least in three replicates. All data analysis and statistical data elaboration were performed using GraphPad Prism, version 8.0 (GraphPad Software, Inc., La Jolla, CA, USA). The program was also used to obtain immunoassay calibration curves by fitting experimental data with both a four-parameter logistic equation (sigmoidal curve) and a logit-log function (linear curve).

### 2.6. Real Sample Processing

The method applicability for the analysis of real samples was assessed by analyzing chocolate chip cookies from different market brands bought in local stores. Sample preparation was carried out according to a previously published procedure [31,32]. Briefly, about 10 g of cookies were grounded with a cooking blender and 1 g of powder was extracted with 10 mL of TRIS buffer. After homogenization by manual shaking, the suspension was shaken for 30 min, then let settle for 5 min. Any upper fat layer was discarded, and the clear supernatant was collected, diluted 1:10 (*v*/*v*) with TRIS buffer and stored at 4 °C in the dark until analyzed.

### 2.7. In Silico Simulations

The molecular modelling of the anti-OVA antibody was based on homology modelling that exploits abYsis, a web-based antibody research system [33,34], and Abymod, an antibody model building tool [35]. In silico binding affinities of proteins with the anti-OVA antibody were calculated by protein-protein molecular docking using HDOCK [36,37] and PRODIGY webservers [38,39]. GROMACS [35,40] was used for structural refinement based on molecular dynamics and energy minimization, while templates for modelling anti-OVA antibody target sequences were obtained from the RCSB Protein Data Bank [41]. The PRODIGY online tool [38] was employed to calculate the thermodynamic binding parameters of complexes between anti-OVA and proteins. The images of complexes were generated by the pyMOL tool [42].

## 3. Results

### 3.1. Synthesis of OVA-MBs

The bioconjugation between MBs and OVA was performed following a previously reported synthetic protocol [43], with slight modifications. Briefly, the surface carboxyl groups of the MBs were activated by a reaction with EDC/sulfo-NHS to produce primary amine-reactive sulfo-NHS esters, which then reacted with OVA to obtain OVA-MBs. The synthetic procedure was optimized to maximize the amount of OVA bound to the MBs, which translated to higher CL signals in the assay.

**Figure 3:**
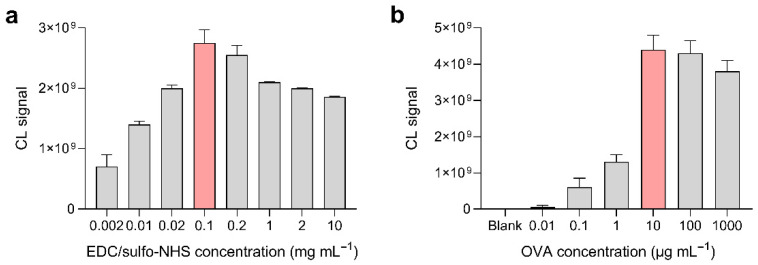
(**a**) CL signals obtained by employing different EDC/sulfo-NHS concentrations for the activation of the surface carboxyl groups of the MBs. Activated carboxyl groups were quantified by reaction with an excess of HRP, followed by CL detection of the bound enzyme. (**b**) CL signals obtained by employing different OVA concentrations in the coating of activated MBs to obtain OVA-MBs. Ovalbumin bound to MBs was quantified by reaction with an excess of anti-OVA-HRP, followed by CL detection of the conjugate. The optimal experimental conditions are highlighted in red. Each of the data are the mean ± SD of three measurements.

First, the concentration of sulfo-NHS and EDC for the activation of the MBs’ carboxyl groups was selected. Different EDC/sulfo-NHS mixtures (1:1 weight ratio) were used to activate the carboxyl groups of MBs. Then, the activated MBs were reacted with a large excess of HRP used as a model protein to verify the efficiency of the activation reaction (HRP was selected as a model, since the amount of HRP bound to the MBs can be easily measured by CL, due to its enzymatic activity). As shown in Figure 3a, the EDC/sulfo-NHS concentration that provided the most efficient activation of carboxyl groups was 0.1 mg mL^−1^ (i.e., 0.1 mg mL^−1^ of EDC and 0.1 mg mL^−1^ of sulfo-NHS). As expected, weaker CL signals, showing an incomplete activation of carboxyl groups, were obtained at lower EDC/sulfo-NHS concentrations. The recorded CL signals were lower also at the highest EDC/sulfo-NHS concentrations, which could be ascribed to the onset of parallel secondary reactions that yielded undesired products, as previously reported by Yan et al. [44]. Then, the best concentration of OVA for the bioconjugation reaction was assessed. Activated MBs were reacted with different concentrations of OVA and the amount of OVA bound to the MBs was measured by CL after incubation of OVA-MBs, with an excess of anti-OVA-HRP. 

As shown in Figure 3b, which reported the CL signal as a function of the OVA concentration used in the coating of MBs, 10 µg mL^−1^ OVA allowed us to achieve the highest CL signals (i.e., the highest amount of OVA bound to MBs and recognized by anti-OVA-HRP). In the absence of OVA, no detectable CL signal was obtained, which confirmed the efficacy of the saturation procedures in avoiding any non-specific binding of immunoreagents to MBs. As can be also observed in Figure 3b, a slight decrease in the CL signal was also observed for the highest OVA concentrations, which should correspond to the greatest OVA loadings. This effect can be attributed to the worse recognition of OVA by the anti-OVA-HRP antibody due to steric hindrance, when a large amount of OVA is immobilized on the MBs surface [45,46].

### 3.2. Design of the Origami µPAD

The origami µPAD consisted of a chromatographic paper sheet in which hydrophilic areas are delimited by wax-printed hydrophobic barriers (the layout of the µPAD allowed four different analyses to be carried out simultaneously). It included the following five functional layers (i.e., A, B, C, D1–D3, E1–E2), each one with a specific function in the assay procedure:

- A: base layer containing OVA-MBs (all (bio)chemical reactions took place in this layer);
- B: anti-leaching layer (a wax-coated sheet that reduced evaporation and prevented solution leaching during incubation);
- C: immunoreaction layer containing the anti-OVA-HRP immunoreagent;
- D1–D3: washing layers (collected the buffer in the washing step);
- E1–E2: CL detection layers containing the luminol/enhancer and sodium perborate CL detection reagents, respectively.

Layers B–E are arranged in a cross shape around the base layer A, thus facilitating their sequential folding during the execution of the steps of the immunoassay protocol (except the first one, each step required folding of only one layer on the base layer). To simplify the assay protocol and eliminate the need for the user to prepare and handle chemicals, all the reagents were preloaded in a dried form on the proper layer. By adding buffers (the only chemicals required for the assay), the dried reagents were dissolved and transported in the base layer, where the (bio)chemical reactions took place. To guarantee fast and uniform migration of solutions between different layers, a holding clip was produced by 3D-printing and used to keep the origami µPAD folded, ensuring close contact between the adjacent layers (a second clip equipped with magnets was used during the loading of OVA-MBs in the origami to avoid their excessive dispersion over the hydrophilic areas).

Incubation and washing steps are more challenging in µPADs as compared with conventional (e.g., microtiter plate-based) immunoassay formats, thus requiring specific design and optimization of the device. The assay relies on binding equilibria that involve both species in solution (i.e., OVA and anti-OVA-HRP) and bound to MBs (OVA) and the incubation step is crucial for obtaining accurate quantitative results. Due to small amounts of reagents and the quite long incubation time, evaporation could significantly reduce the volume of solution during incubation, thus altering the concentration of chemicals and affecting the binding equilibria. The stacking of layers C, A and B (from top to bottom) of the origami µPAD during the incubation created a well-like structure, in which the hydrophilic areas of layers A and C constituted the “well” volume, and the hydrophobic layer B acted as the “well” bottom. This accommodated the solution, avoiding leaking and reducing evaporation, which could only take place at the surface of layer C. Effective washing is also critical in immunoassays because incomplete removal of excess reagents and of non-specifically bound species greatly affects assay sensitivity and reproducibility. The latter phenomenon is particularly important in µPAD-based formats, since the interaction between biomolecules and cellulose fiber can lead to nonspecific adsorption phenomena, especially for polar or charged molecules [21]. Furthermore, due to capillarity effects of paper, the complete removal of the washing solution is difficult; thus, for efficient washing, a high and sustained flow of liquid across paper is needed. It has been previously shown that this can be obtained in a µPAD geometry that would provide a steady increase in the available wettable volume. This approach, which was described for a 2D planar configuration [47], was adapted in this work to a 3D geometry by designing a device in which three paper layers (D1–D3) provided, once folded over layer A, circular hydrophilic crowns with increasing diameter (from 6 to 9 mm). With this configuration, the washing buffer flows both vertically across the different folded layers and radially towards the boundary hydrophilic zone [48,49,50]. In addition, the layers D1–D3 had a central hole (4 mm diameter) to avoid any mechanical loss of OVA-MBs when unfolding the origami due to the contact with the layer D1.

Theoretical approaches for modelling flow in the paper substrate have been proposed to accelerate development of µPADs [51]. The most applied ones rely on the Lucas–Washburn equation [52] or the Darcy’s law [53]. However, they only are suited for nearly bidimensional paper-based systems (e.g., single-layer paper devices) and simple geometries [54,55]. Fluid dynamics in a 3D paper-based device is of greater complexity and its theoretical treatment requires understanding of the physics that regulates microfluidics, as well as of the influence of several variables, in addition to the fluidics geometry, such as the characteristics of porous material and the type, ionic strength, and viscosity of the fluid [56,57].

In this paper, we adopted a simple approach to investigate the effect of D1–D3 layers’ geometry on the fluid motion. We used a mathematical model that described the trajectory of the liquid flow from the detection zone in layer A towards the washing layers as a function of their geometry, considering only the direction of the contours of each layer. This model is based on the following hypotheses, which described an ideal behavior [58,59]: (a) the fluid is assumed to be non-viscous, neglecting internal friction; (b) the fluid is incompressible; (c) the motion of the flow is stationary (i.e., its velocity at each point does not change); (d) the flow is irrotational (i.e., the angular momentum of the fluid is zero at any point). The three-dimensional trajectory of the liquid was described according to a system of parametric equations in polar coordinates of the general form *h* = *f*(*u*,*t*) (*h* = *i*-th component of the position vector; *u*, *t* = polar coordinates).

(1) $$\{\begin{array}{c} x\left(t,u\right)=\left(n_{1}+nt^{0.7}\right)cos\left(2u\right)\\ y\left(t,u\right)=\left(n_{1}+nt^{0.7}\right)sin\left(2u\right)\\ z\left(u\right)=-n_{2}sin\left(n_{3}t\right) \end{array}\;\;\;\;n_{i}>\;0;\;0\;<\;t\;<\;2\;\pi ;\;0\;<\;u\;<\;\pi$$

**Figure 4:**
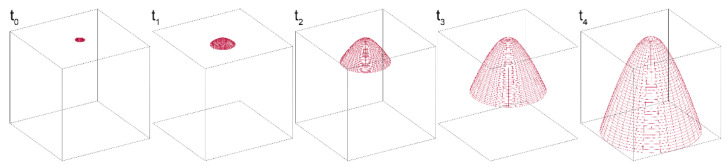
Simulation of 3D trajectory of fluid across the washing layers D1–D3 at various times (*t*_0_ > *t*_1_ > *t*_2_ > *t*_3_ > *t*_4_). The fluid trajectory was described according to a system of parametric equations in polar coordinates (Equation (1)), considering only the direction of the contours of the fluid in each layer. A bell-shaped trajectory is obtained for the fluid front moving across the D layers, characterized by the increasing diameter of the hydrophilic area. This can be ascribed to the combination of a radial movement towards the boundary of the hydrophilic zone of a given D layer and a vertical movement between the adjacent D layers.

The results reported in Figure 4 showed that the flow of the liquid passing from layer A to the washing layers D1–D3 followed a bell-shaped trajectory, assuming the shape of crowns with rays of increasing size for the successive layers. Therefore, the design of the µPAD with an increasing radius of the hydrophilic zones of layers D1–D3, which made available a larger wettable volume for the fluid when it moved vertically through the layers, eased the flow of liquid (thus the removal of unbound species) from the hydrophilic areas of layer A.

### 3.3. Optimization of the Origami µPAD and Assay Performance

**Figure 5:**
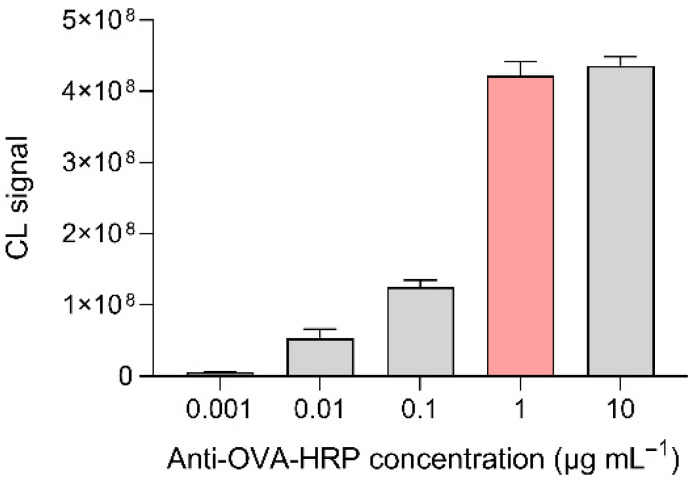
CL signals obtained by analyzing OVA-free solutions (PBS) in origami µPADs prepared using anti-OVA-HRP solutions at different concentrations. The optimal anti-OVA-HRP concentration is highlighted in red. Each of the data are the mean ± SD of three measurements.

While the CL detection reagents should be present in large excess, the amount of anti-OVA-HRP conjugate loaded in the origami µPAD is crucial for assay performance. Indeed, to achieve the best assay performance in terms of limit of detection (LOD), the anti-OVA-HRP should be just sufficient to saturate the binding sites on MBs (an excess of anti-OVA-HRP shifted the assay calibration curve towards higher concentrations, thus increasing the LOD). To determine the best amount of anti-OVA-HRP, we analyzed OVA-free solutions (PBS) in origami µPADs, prepared by loading anti-OVA-HRP solutions at different concentrations, and measured the resulting CL signals. Figure 5 showed that the CL signal (thus the amount of anti-OVA-HRP bound to the MBs) increased for anti-OVA-HRP solution concentrations up to 1 µg mL^−1^, then remained nearly constant. Based on this result, this concentration was selected for the preparation of the µPADs.

**Figure 6:**
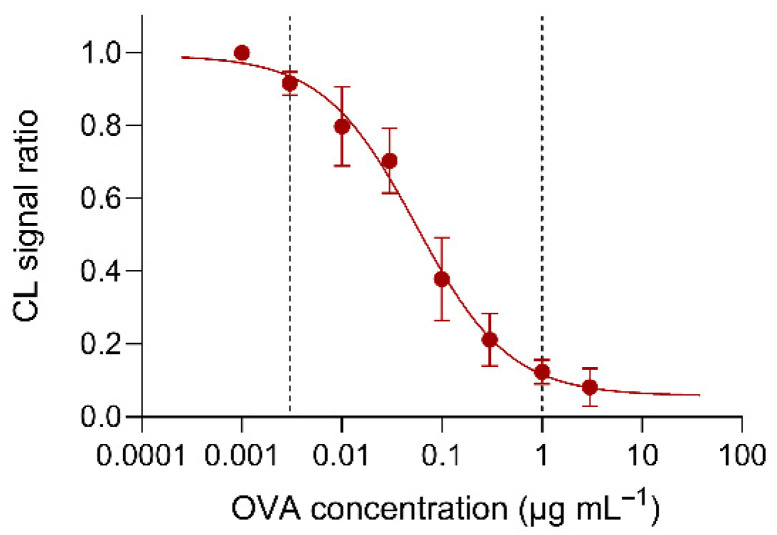
Calibration curve generated by combining the results obtained by analyzing OVA standard solutions with different biosensors. A four-parameter logistic equation was used to fit the experimental data and the equation of the resulting calibration curve was Y = 0.925/(1 + 10(0.932(1.257 + X))) + 0.057 (R2 = 0.994), where Y and X were the CL signal ratio and the logarithm of concentration of OVA standard solutions. The dashed lines show the assay range (see text). Each of the data are the mean ± SD of three measurements.

Figure 6 shows the calibration curve generated in the optimized experimental conditions, obtained by plotting the ratios between the CL signals measured for different OVA standard solutions and the signal measured in the absence of OVA (i.e., the immunoassay B/B_0_ parameter) against the logarithm of OVA concentration. Since the number of samples that can be analyzed in an origami is limited, the calibration curve has been obtained by combining the results of several biosensors (three OVA standard solutions were assayed in each µPAD, together with an OVA-free sample, then the B/B_0_ parameter for each standard solution was calculated and the data from different µPADs were joined). A four-parameter logistic equation was used to fit the experimental data and obtain the calibration curve parameters. According to the equation of the calibration curve, the LOD of the assay (calculated as the concentration of OVA corresponding to the CL signal of the OVA-free sample, minus three times its standard deviation) was 1 ng mL^−1^, while the assay working range (estimated as the range of OVA concentrations that correspond to the 10-to-90% change in the CL signal ratio) was from 0.003 to 1 µg mL^−1^ of OVA.

### 3.4. Measurement of OVA with the Origami µPAD

**Figure 7:**
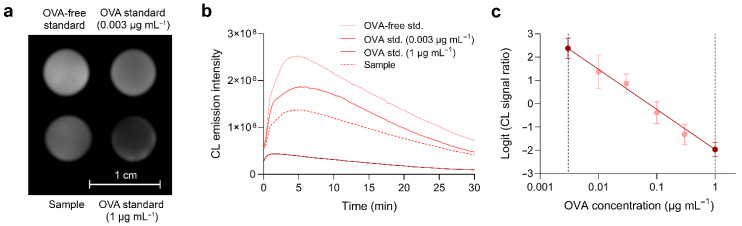
(**a**) CL image of the origami µPAD acquired during the assay. The scale bar represents 1 cm. (**b**) CL emission intensity kinetic profiles obtained by the analysis of the CL images acquired during the assay. (**c**) Application of the two-standard calibration approach to the calibration data of Figure 6. The readings obtained for concentrations that correspond to the upper and lower limits of the assay working range (dark points) were used to obtain the two-point calibration curve, while the other readings (light points) were simply plotted on the graph.

Since the number of samples that can be analyzed in a single origami µPAD is limited, an analytical procedure that relies on a multiple-point calibration curve could be only performed using several origami devices, which would complicate assay execution. To perform the assay in a single µPAD, we developed a procedure that requires the analysis of the sample and of three standards, i.e., an OVA-free solution (PBS) and two OVA standards in PBS at concentrations that correspond to the upper (1 µg mL^−1^) and lower (0.003 µg mL^−1^) limits of the assay working range. After evaluation of the ratios between the CL signals of the OVA standards and of the OVA-free solution, a two-point linear calibration curve was obtained by plotting the logit of the CL signal ratios of the OVA standards against the logarithm of OVA concentrations (such a procedure is often used to linearize the central portion of the sigmoidal calibration curves of competitive immunoassays) and the amount of OVA in the unknown sample was evaluated by interpolation of its CL signal ratio logit on the linear calibration curve. No blank was needed for this procedure, since the non-specific binding of anti-OVA-HRP was negligible (we verified this in origami µPADs prepared with MBs conjugated with BSA, instead of OVA). In case many samples need to be analyzed, the assay could be also conducted by using an origami to produce the calibration curve and then employing other origami µPADs to analyze four samples at the same time (the inter-origami variability in the CL signals measured in origami µPADs from the same production lot was less than 5%). Figure 7a,b show, respectively, a representative CL image of the origami µPAD and the CL emission intensity kinetic profiles, obtained by the quantitative analysis of the sequence of CL images acquired during the assay. To demonstrate the feasibility of the two-standard calibration approach, we applied such an approach to the data of the calibration curve of Figure 6. Figure 7c shows the two-point linear calibration curve obtained from the points at the upper and lower limits of the assay working range, while the other points within the working range were simply plotted on the graph. All the intermediate calibration points are on the calibration curve; therefore, this approach can be used to calculate the OVA concentration of unknown samples within the assay working range.

### 3.5. Assay Specificity

**Figure 8:**
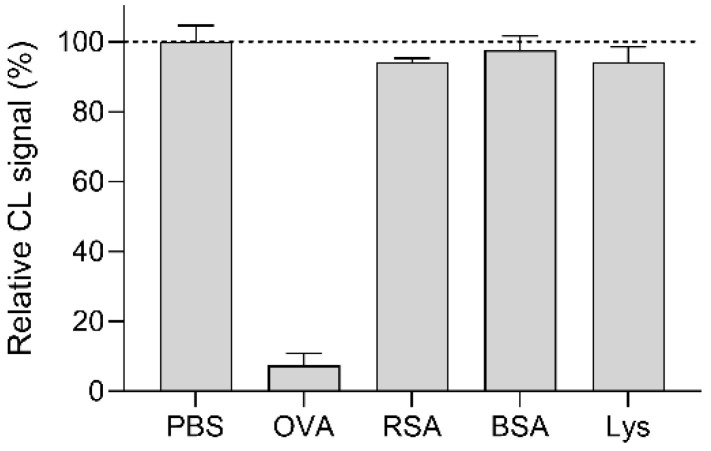
CL signals measured in the origami µPAD for an OVA-free standard solution (PBS) and 10 µg mL^−1^ standard solutions of the potentially interfering proteins RSA, BSA, and Lys. For comparison, the CL signal measured for a 10 µg mL^−1^ OVA standard solution is reported. Each of the data are the mean ± SD of three measurements.

To evaluate assay specificity, standard solutions of other proteins commonly found in food, such as bovine serum albumin (BSA), rabbit serum albumin (RSA), and lysozyme from chicken egg white (Lys), were analyzed with origami µPADs. Figure 8 shows the comparison between the CL signals measured for 10 µg mL^−1^ standard solutions of the potentially interfering proteins and an OVA-free standard solution (i.e., PBS), for which the largest amount of anti-OVA-HRP was bound to the OVA-MBs. The results showed that, even at such relatively high concentrations, Lys, RSA, and BSA did not significantly interfere with the binding of anti-OVA-HRP to OVA-MBs (for comparison, a 10 µg mL^−1^ OVA standard solution displaced about 95% of the anti-OVA-HRP conjugate from the OVA-MBs).

For a better comprehension of assay specificity, an in-silico model was used to compare the experimental results with computational data. Computational modelling methods for the in-silico construction of the 3D structure of monoclonal antibodies (mAbs) have been already developed and applied in drug discovery [60,61], while their exploitation to support immunosensor development is not yet widespread. Furthermore, many immunosensors employ polyclonal antibodies (pAbs) rather than mAbs, taking advantage of their higher binding avidity and affinity [62,63]. However, pAbs are heterogeneous mixtures; thus, developing a theoretical model of antigen–antibody interaction in the case of pAbs would require high computational effort. To overcome this limitation, the in-silico model proposed in this manuscript used complementary computational techniques to simulate approximate models of antibody–antigen complexes. In our opinion, the use of an approximate model that is capable of simulating, in a simple manner, an ideal case in which the interaction between biological macromolecules is optimal does not represent a limitation. In fact, theoretical models based on mathematical approximations are commonly employed in other fields of chemistry, such as investigation of reaction mechanisms [64,65,66] or simulation of spectroscopy experiments [67].

**Figure 9:**
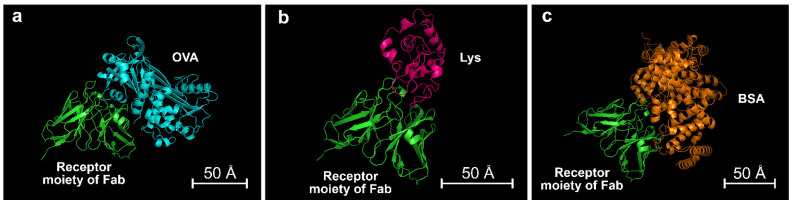
Most probable 3D structures obtained by molecular docking simulations for the complexes of (**a**) OVA, (**b**) Lys, and (**c**) BSA with anti-OVA. The scale bars represent 50 Å.

In detail, the model was used to calculate the thermodynamic stability parameters of the antibody–antigen complexes formed between the anti-OVA antibody and either OVA or the tested interfering proteins. Due to the absence of a crystallographic structure for the anti-OVA antibody, its modelling was performed starting from sequences obtained from the abYsis webserver [33,68]. In particular, the sequences of the variable regions in the heavy (V_H_) and light (V_L_) chains of the Fab fragment of anti-OVA mAbs produced by a hybridoma were employed. The chosen sequences were subjected to homology modelling in the AbYmod webserver, which automatically selected the most suitable PDB templates from the Protein Data Bank for modelling the target sequence. Structural refinement based on molecular dynamics and energy minimization were performed in GROMACS, which provided the 3D molecular structure of the Fab fragment of anti-OVA. The final model was employed in ab initio protein-protein molecular docking simulations in the presence of either the target protein (1OVA.pdb) or interfering proteins [36,69] to generate the most plausible, lower-energy complexes, based on topological and electrostatic complementarity (Figure 9). Finally, the binding stability of the complexes anti-OVA-OVA, anti-OVA-Lys, and anti-OVA-BSA was evaluated using the PRODIGY online tool to obtain the corresponding value of ΔG_bind_. The results of the molecular docking simulations (Table 1) showed that, in agreement with the experimental results, the antibody has a higher affinity for OVA. The stability of the anti-OVA-Lys complex is much lower and the anti-OVA-BSA complex is even less stable, thus confirming the antibody specificity. We suggest that in-silico modelling of biomolecule interaction can be a useful tool for supporting biosensor development and, when needed, aid in the optimization of experimental parameters, such as buffer, pH, and ionic strength, to improve assay analytical performance.

### 3.6. Accuracy and Quantification of Ovalbumin in Real Samples

**Figure 10:**
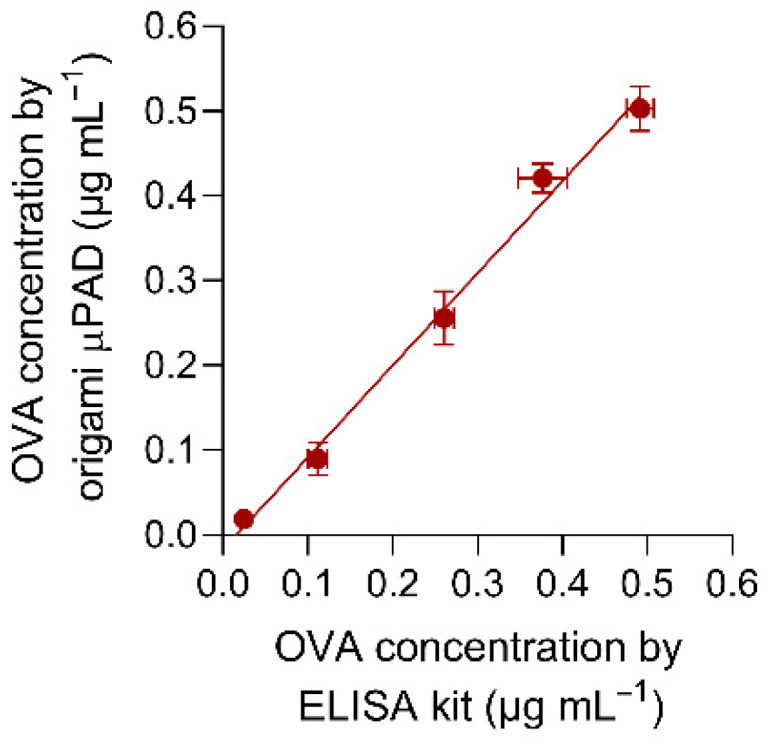
Comparison between OVA concentrations measured in real samples by using the origami µPAD and the colorimetric ELISA kit reference method. The equation of the linear regression curve is Y = 1.087 X − 0.018 (R^2^ = 0.992), where Y and X are the OVA concentrations measured with the origami µPAD and the colorimetric ELISA kit reference method, respectively. Each of the data are the mean ± SD of three measurements.

The accuracy of the origami µPAD biosensor for OVA was evaluated by comparison of measurements obtained for real samples (chocolate chip cookies from different market brands bought in local stores). All samples were analyzed both with the origami µPAD and with a colorimetric ELISA kit reference method, based on a non-competitive sandwich immunoassay using anti-OVA-HRP. The results reported in Figure 10 showed a good correlation between the two methods (R^2^ > 0.98).

We also investigated the potential interference of a real sample matrix by performing a recovery study. An extract of chocolate chip cookies (obtained as described in Section 2.6) with OVA content below the LOD of the assay was spiked with known amounts of OVA and analyzed using the origami µPAD. A previously reported procedure for sample extraction was employed, in which a 0.025 M TRIS buffered solution was employed to ensure analyte recovery from the matrix at controlled pH. In case of highly acidic food samples, a TRIS buffer with higher buffering capacity might be employed for sample extraction. As shown in Table 2, we obtained good correspondence between the OVA concentrations measured in spiked extracts and the added OVA. Recovery ranged from 83 to 133%, which can be considered adequate for a point-of-use immunosensor, as compared with the 80–120% recovery commonly accepted for laboratory immunoassays [70]. The assay precision for the real samples was also found to be satisfactory for a point-of-use assay, with a coefficient of variation below 15%. Overall, these results proved the effectiveness of the origami µPAD for the analysis of OVA in the real samples tested.

### 3.7. Stability of the Origami µPAD

The stability over time of the reagents (OVA-MBs, anti-OVA-HRP, luminol/enhancer, and sodium perborate) loaded in the origami µPAD has been investigated. A series of origami µPADs loaded only with the reagent under study was sealed under a vacuum in plastic bags and stored at 4 °C. After a given storage time (up to 6 weeks), the remaining reagents were loaded in the µPAD, then the CL signals obtained for the analysis of an OVA-free standard were measured. The comparison of the CL signals with that obtained in a freshly prepared µPAD allowed us to assess the degradation over time of the investigated reagent.

**Figure 11:**
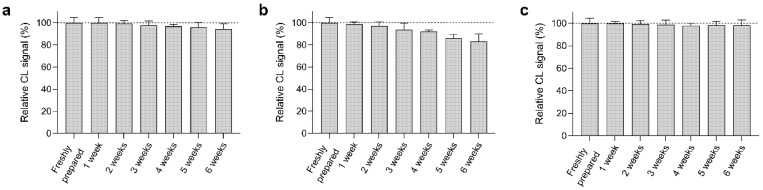
Decrease in the CL signal measured upon storage at 4 °C for origami µPADs containing the reagents (**a**) OVA-MBs, (**b**) anti-OVA-HRP, and (**c**) luminol/enhancer and sodium perborate. The remaining reagents were loaded in the µPADs just before the measurement. Each of the data are the mean ± SD of three measurements.

As shown in Figure 11, OVA-MBs, luminol/enhancer, and sodium perborate displayed good stability, with a CL signal decrease of less than 10% after 6 weeks of storage. On the other hand, the anti-OVA-HRP conjugate markedly decreased its bioactivity (up to 40% after only 3 weeks of storage, data not shown). To improve the stability of the conjugate, we added the low molecular weight polyol sorbitol to the anti-OVA-HRP solution loaded in the µPAD [71]. This significantly increased the stability of the conjugate and made it possible to use the ready-to-use µPAD after up to 4 weeks of storage. We also investigated the use of other protein protecting agents, such as pullulan, a polysaccharide that ensures high protein stability upon drying [72]. Unfortunately, pullulan negatively affected the re-solubilization of anti-OVA-HRP upon addition of the buffer, thus making the assay unfeasible.

## 4. Conclusions

This article describes an origami µPAD for the quantitative determination of OVA in food samples that displayed improved performance and were suitable for on-site application. The assay relied on a competitive immunoassay, followed by CL detection by a luminol/hydrogen peroxide system. The use of magnetic microbeads allowed easy and efficient immobilization of immunoreagents in the µPAD. Due to the origami approach, it was possible to fully implement on paper a multi-step analytical procedure and to avoid chemical handling by the operator, as all the reagents were preloaded in the µPAD. The assay proved to be suitable for the detection of OVA traces in real samples in a relatively short period of time (i.e., approximately 1 h). The same approach could be used for other allergens or clinical protein markers. Future work is foreseen to evaluate the use of a smartphone’s camera and a detector in the substitution of the portable CCD and dedicated application for data elaboration, to further improve assay portability and widespread applicability [73].

## Figures and Tables

**Table 1:** Predicted binding affinities and dissociation constants of complexes between anti-OVA and target proteins ^1^.

Target Protein	Binding Affinity (ΔG_bind_) (kcal mol^−1^)	Dissociation Constant (K_d_) (mol L^−1^)
OVA	−12.3	8.9 × 10^−10^
Lys	−10.9	1.1 × 10^−8^
BSA	−9.1	2.1 × 10^−7^

^1^ RSA was highly similar (>90%) in sequence with BSA; thus, its binding affinity to anti-OVA was not evaluated.

**Table 2:** Results of the recovery study of the origami µPAD biosensor performed on a blank extract of biscuit sample spiked with known amounts of OVA.

	Concentration of OVA Spiked (µg mL^−1^)	Concentration of OVA Measured (µg mL^−1^)	Recovery (%)
Sample 1	0.003	0.004	133
Sample 2	0.010	0.009	89.2
Sample 3	0.020	0.025	124
Sample 4	0.100	0.083	82.6
Sample 5	0.400	0.383	95.8

## Data Availability

Not applicable.

## Supplementary Materials

The following supporting information can be downloaded at: https://www.mdpi.com/article/10.3390/bios12100825/s1, Video S1: Assay procedure.
